# Predicting chemotherapy toxicity in multiple myeloma: the prognostic value of pre-treatment serum cytokine levels of interleukin-6, interleukin-8, monocyte chemoattractant protein-1, and vascular endothelial growth factor

**DOI:** 10.3389/fimmu.2024.1377546

**Published:** 2024-05-23

**Authors:** Michał Mielnik, Martyna Podgajna-Mielnik, Aneta Szudy-Szczyrek, Iwona Homa-Mlak, Radosław Mlak, Aneta Gorący, Marek Hus

**Affiliations:** ^1^ Department of Hematooncology and Bone Marrow Transplantation, Medical University of Lublin, Lublin, Poland; ^2^ Department of Human Physiology, Medical University of Lublin, Lublin, Poland; ^3^ Department of Laboratory Diagnostics, Medical University of Lublin, Lublin, Poland

**Keywords:** multiple myeloma, chemotherapy, interleukins, cytokines, toxicity, IL-6, IL-8, MCP-1

## Abstract

**Introduction:**

Multiple Myeloma (MM), a prevalent hematological malignancy, poses significant treatment challenges due to varied patient responses and toxicities to chemotherapy. This study investigates the predictive value of pretreatment serum levels of interleukin-6 (IL-6), interleukin-8 (IL-8), monocyte chemoattractant protein-1 (MCP-1), and vascular endothelial growth factor (VEGF) for chemotherapy-induced toxicities in newly diagnosed MM patients. We hypothesized that these cytokines, pivotal in the tumor microenvironment, might correlate with the incidence and severity of treatment-related adverse events.

**Methods:**

We conducted a prospective observational study with 81 newly diagnosed MM patients, analyzing serum cytokine levels using the multiplex cytometric bead assay (CBA) flow cytometry method. The study used non-parametric and multivariate analysis to compare cytokine levels with treatment-induced toxicities, including lymphopenia, infections, polyneuropathy, and neutropenia.

**Results:**

Our findings revealed significant associations between cytokine levels and specific toxicities. IL-8 levels were lower in patients with lymphopenia (p=0.0454) and higher in patients with infections (p=0.0009) or polyneuropathy (p=0.0333). VEGF concentrations were notably lower in patients with neutropenia (p=0.0343). IL-8 demonstrated an 81% sensitivity (AUC=0.69; p=0.0015) in identifying infection risk. IL-8 was an independent predictor of lymphopenia (Odds Ratio [OR]=0.26; 95% Confidence Interval [CI]=0.07-0.78; p=0.0167) and infection (OR=4.76; 95% CI=0.07-0.62; p=0.0049). High VEGF levels correlated with a 4-fold increased risk of anemia (OR=4.13; p=0.0414).

**Conclusions:**

Pre-treatment concentrations of IL-8 and VEGF in serum can predict hematological complications, infections, and polyneuropathy in patients with newly diagnosed MM undergoing chemotherapy. They may serve as simple yet effective biomarkers for detecting infections, lymphopenia, neutropenia, and treatment-related polyneuropathy, aiding in the personalization of chemotherapy regimens and the mitigation of treatment-related risks.

## 1 Introduction

Multiple myeloma (MM), the second most prevalent hematological malignancy, presents a complex clinical landscape marked by the aberrant proliferation of plasma cells and the production of dysfunctional monoclonal proteins (1). This disease predominantly afflicts older adults, with the average age of onset at 70 years, and is propelled by an array of chromosomal aberrations and genetic mutations that culminate in the expansion of a malignant plasma cell clone (2, 3). The insidious onset of MM, often heralded by nonspecific symptoms such as weakness, weight loss, and bone pain, can delay diagnosis until the emergence of more severe complications like disseminated bone lesions, pathological fractures, bone marrow failure, and renal damage, which are an indication for treatment (4, 5).

Newly diagnosed MM patients (NDMM) considered fit (aged <70 years, without comorbidities) are recommended to receive induction followed by high-dose therapy (HDT) with autologous hematopoietic stem cell transplantation (auto-HSCT) and lenalidomide maintenance (6). The induction treatment is usually based on the bortezomib and dexamethasone (Vd) backbone. The updated 2021 European Hematology Association (EHA) and European Society for Medical Oncology (ESMO) clinical practice guidelines recommend the use of either daratumumab-thalidomide-Vd (Dara-VTd) or lenalidomide-Vd (VRd) as first-line options (7).

Elderly patients or patients with NDMM who are not eligible for auto-HSCT should receive VRd (bortezomib, lenalidomide, dexamethasone), DaraVMP (Daratumumab, bortezomib, melphalan, dexamethasone) and DaraRd (Daratumumab, lenalidomide, dexamethasone) (7, 8).

While advancements in treatment, including the use of proteasome inhibitors, immunomodulatory drugs, and monoclonal antibodies, have significantly improved survival outcomes, with a median survival rate that now exceeds six years, with some subsets of patients surviving more than eight years, MM remains incurable (9–12). However, introducing innovative MM treatment, better diagnostics, and improved medical care make the expected OS of patients diagnosed today almost impossible to assess. The newest developments in antibody-drug conjugates, bi-specific antibodies, or chimeric antigen receptor T-cell therapy (CAR-T) cell therapies change the prognosis entirely, even for patients with relapsed and refractory MM (RRMM) (13).

As the therapeutic arsenal against MM expands, so does the imperative to manage treatment-related toxicities, which can range from hematological and gastrointestinal effects to cardiotoxicity and neuropathies (14–16). These toxicities not only compromise patient quality of life but can also necessitate dose reductions, potentially diminishing the efficacy of the therapeutic regimen (17). However, some patients are more prone to selected toxicities (18). The reason for that is often unknown. The ability to find such patients even before starting the therapy could influence the therapy choice, thus significantly improving its outcomes and safety.

Within the intricate milieu of the MM microenvironment, soluble mediators such as cytokines assume pivotal roles in orchestrating immune responses that are fundamental to both the pathology of the disease and the patient’s response to chemotherapy.

Our study is motivated by the pivotal role that cytokines within the MM microenvironment play in tumor growth, angiogenesis, and the immune response (19, 20). The cytokines of interest in this study—IL-6, IL-8, MCP-1, and VEGF—have been selected for their prominence in the MM microenvironment and their documented influence on disease progression and response to treatment, as well as their less understood roles in mediating chemotherapy-induced toxicity.

IL-6, a cytokine with both pro- and anti-inflammatory actions, has been implicated in the pathogenesis of MM, with elevated levels noted in many patients (20–22). It acts as a growth factor for myeloma cells, and its influence on angiogenesis suggests a role in disease advancement. Similarly, IL-8, a pro-inflammatory chemokine, is involved in angiogenesis and may contribute to MM progression and metastasis (19, 23–26). Its regulatory effect on myelopoiesis also presents a potential mechanism through which it could influence treatment responses (27, 28).

VEGF, a key mediator of angiogenesis, has been associated with more aggressive MM variants and may also modulate responses to novel anti-MM agents with antiangiogenic properties (29–31).

Lastly, MCP-1’s role as a chemoattractant and its involvement in angiogenesis and osteoclastogenesis suggest it may be pivotal in MM pathophysiology and associated bone disease (32–34).

Our research addresses the critical need to understand how these soluble factors can be leveraged to predict and potentially mitigate the toxicities associated with MM treatment. By situating these cytokines within the context of immune cell recruitment, activation, and inflammation resolution. Incorporating our previous research findings (35), this study utilizes a multiplex cytometric bead assay to investigate pretreatment serum levels of these cytokines as potential predictors of chemotherapy-induced toxicity. By exploring these associations in a cohort of 81 newly diagnosed MM patients, we aim to contribute to the personalization of treatment modalities, potentially improving therapeutic outcomes and patient well-being.

## 2 Materials and methods

### 2.1 Ethical statement

This study received approval from the Medical University of Lublin’s Ethics and Research Committee (consent no.: KE-0254/26/2015), aligning with the ethical standards laid out in the Declaration of Helsinki. Written informed consent was obtained from each participant, detailing the study’s scope, data usage, and the rights to withdraw consent without prejudice. Voluntary participation and rigorous data protection protocols underscore our commitment to ethical research practices.

### 2.2 Study group

The cohort under study included 81 patients diagnosed with multiple myeloma (MM), with 50.6% being male and a median age of 65 years (ranging from 56 to 74 years). Diagnosis was established upon the identification of at least 10% clonal plasma cells in bone marrow biopsies, alongside one or more myeloma-defining events (MDE) according to the SLiM CRAB criteria. This encompasses clonal bone marrow plasma cell levels of 60% or higher, a serum free light chain (FLC) ratio of 100 or more, and the presence of more than one focal lesion on MRI, in addition to the conventional indicators such as hypercalcemia, renal failure, anemia, or lytic bone lesions (5). We relied on Durie-Salomon (36) and International Staging System (ISS) (37) classification for disease staging, while patient performance status was gauged according to the Eastern Cooperative Oncology Group - World Health Organization (ECOG) guidelines (38). We measured the response to treatment using the current International Myeloma Working Group guidelines (39).

The study’s cohort composition was exclusively white individuals not of Hispanic or Latino origin, mirroring the demographic makeup of the Polish population, which lends specificity and relevance to the findings within this healthcare context.

The control group included 49 healthy counterparts, carefully selected to match the primary group’s age and gender distribution.

First-line regimens consisted of thalidomide and/or bortezomib combined with steroids and/or cyclophosphamide and represented a standard of care in Poland during the recruitment period. The chemotherapy regimens chosen for patients in this study were selected based on a multifaceted assessment of each patient’s individual clinical profile. This tailored approach considered the availability and reimbursement for therapies at that time, the latest consensus guidelines, patient age and health status, specific genetic and molecular characteristics of the myeloma, and the need to balance efficacy with potential side effects. Each regimen was selected with the intention of optimizing therapeutic outcomes while considering the tolerability and patient-specific factors that could impact treatment response.

### 2.3 Inclusion and exclusion criteria for the study

We established inclusion and exclusion criteria to ensure a uniform study population that would be most appropriate for examining the specific research question of predicting chemotherapy toxicity through pre-treatment cytokine levels. We chose parameters to ensure that our findings would directly apply to the typical MM patient population treated at our institution. Eligible patients were those with treatment-naive MM diagnosed at our institution within the recruitment timeframe, who required initialization of therapy, and who could provide informed consent (40). We did not include patients with Smoldering Multiple Myeloma (SMM) and Monoclonal Gammopathy of Undetermined Significance (MGUS).

Exclusion criteria were strategically set to mitigate confounding factors: a history of systemic cancer treatment within two years, the presence of autoimmune diseases, or ongoing infections—all known to influence cytokine profiles significantly, thereby potentially skewing the data related to proinflammatory cytokines and their role in treatment-induced toxicity (41, 42).

### 2.4 Data collection

We adopted an observational study design, allowing us to monitor the standard-of-care therapies as administered and to collect clinical data prospectively without influencing therapeutic choices. Our approach ensured the inclusivity of various frontline MM treatments, thereby enhancing the applicability of our findings to evolving therapeutic paradigms. The cytokine levels were measured at diagnosis before the commencement of any treatment to serve as a baseline for subsequent toxicity correlations. During chemotherapy (CTH), we monitored patients for treatment-induced toxicity (hematological and non-hematological toxicities, including infections, polyneuropathies, thromboembolic events, diarrhea, constipation, and other less frequent (reported in the article as “Other toxicities”): acute kidney damage, epistaxis, electrolyte disturbances, nausea, vomiting, fatigue, dizziness, hyperglycemia, mental disorders, oedemas, cardiological disorders, arrhythmias, paresis of the lower limbs, stomach ulcer disease, hypogammaglobulinemia, osteonecrosis of the mandible and maxilla, bowel obstruction, rash, allergic reaction to the drug). The toxicities were tested based on the laboratory results, physical examination, detailed anamnesis, and additional diagnostic procedures if required (e.g., neurological assessment, ECG, gastroscopy). Follow-up assessments for toxicity were conducted in accordance with our treatment protocols. We have documented the incidence and nature of toxicities associated with each CTH regimen used in the study cohort.

We systematically evaluated the toxicities based on CTCAE v 5.0 guidelines, ensuring a standardized assessment approach (43). Wherever in the study the kidney function is referred to as “A” or “B,” it means the creatinine level was < or ≥ 2.0mg/d, respectively.

### 2.5 Study material

We collected approximately 4.5 ml of peripheral blood into serum gel tubes from each participant at diagnosis immediately before treatment initiation. To minimize potential bias, we anonymized samples with a unique four-digit code. Following a standardized centrifugation protocol (3000 rpm for 10 min), the serum was aliquoted and preserved at –80°C, ensuring sample integrity for subsequent cytokine analysis. Patients were recruited in the Department of Hematooncology and Bone Marrow Transplantation in Lublin between 2015 and 2019. The follow-up period spanned 2015–2021, providing a substantial temporal framework for the study.

### 2.6 Cytokine and chemokine measurement

The Cytometric Bead Array (CBA) Solutions is a flow cytometry method used to measure a variety of soluble and intracellular proteins, including cytokines, chemokines, growth factors, and phosphorylated signaling proteins. We employed the CBA technique, leveraging its ability to quantitatively analyze multiple cytokines and chemokines in a single sample. This method is compared favorably to traditional ELISA and Western blot methods regarding efficiency and sample economy (44). Many studies have shown a good correlation between results obtained with CBA and ELISA (45–47).

In our study, we employed a flow cytometry-based multiplex method using microspheres of 7.5 μm diameter, tagged with various fluorescence intensities and antibodies to capture specific analytes. This allowed for the simultaneous measurement of different cytokines in a single sample. We employed the BD CBA Human Flex Sets for IL-6, IL-8, MCP-1, and VEGF, together with the corresponding master buffer kits, and analyzed them with a BD FACSCanto™ II flow cytometer, using FCAP Array™ Software for data interpretation (Becton Dickinson, USA). Recombinant protein standards were integrated into each assay, serving both as internal controls and as a basis for generating standard curves for quantitative analysis. The sensitivity range for the BD CBA Human Enhanced Sensitivity Flex Set spanned from 274 fg/mL to 200,000 fg/mL. For the BD CBA Human Soluble Protein Flex Set, the concentration range was specified from 10 pg/mL to 2,500 pg/mL. Theoretical detection limits were established for IL-6 at 68.4 fg/mL, IL-8 at 69.9 fg/mL, MCP-1 at 1.3 pg/mL, and VEGF at 4.5 pg/mL. This was achieved by evaluating the negative controls’ mean fluorescence intensity (MFI), adjusted for 30 iterations of each set plus two standard deviations. Serum samples were diluted 1:4 to match standard curve ranges.

Following the manufacturer’s instructions, we reconstituted and serially diluted the lyophilized BD CBA Human Flex Set Standards immediately before mixing with the Capture Beads and the Detection Reagent. First, we ran the standards from least concentrated (0 fg/mL or 0 pg/ml, respectively) to most concentrated (Top Standard) to facilitate analysis in FCAP Array software. Then, we diluted the capture beads to their optimal concentrations.

A two-step detection system was utilized for the enhanced sensitivity sets, involving a mix of detection reagents and a subsequent addition to the assay tube after reconstitution and dilution.

Instrument calibration was performed before each run with calibration beads to ensure accurate readings. Analytes from the samples formed complexes with the capture beads and detection reagents, identifiable by their dual-fluorescence signature, enabling precise quantification of cytokine concentrations. The intensity of PE fluorescence of each sandwich complex is directly proportional to the analyte’s concentration within the sample.

Samples were analyzed immediately after preparation to maintain sensitivity, with at least 5,000 events per analyte captured. Post-acquisition, data were processed using FCAP Array™ software, which produced both graphical and tabular representations of cytokine concentrations, as illustrated in **Supplementary Figure 1**.

### 2.7 Statistical analysis

We employed MedCalc software (version 15.8 PL) and Statistica (version 13 PL) for statistical analysis. Categorized or dichotomized variables were expressed in absolute numbers and percentages. To evaluate the normality of continuous data distribution, we applied the D’Agostino-Pearson test. Given that our continuous variables were not normally distributed, we used the median, interquartile range, and range (minimum-maximum) as dispersion measures and applied non-parametric tests accordingly. The Mann-Whitney U test assessed the differences in cytokine concentrations in relation to specific treatment-induced toxicities. We used Spearman’s rank correlation test to determine the relationship between cytokine levels and the severity of treatment-related toxicities. The Receiver Operating Characteristic (ROC) curves were instrumental in evaluating the diagnostic accuracy of the cytokines for predicting specific toxicities. In our report, “high” and “low” are relative terms referring to concentrations above or below the median, respectively.

The sample size calculations were based on previously published data regarding IL-8 serum concentration in MM patients (37.7 + 13.5 pg/ml) and healthy controls (28.7 + 7.6 pg/ml). The ratio of sample sizes in the compared groups was equal to 1:2.65 (48). Setting a value of 0.05 for type I errors (alpha) and 0.01 (allowing for the achievement of nearly 100% statistical power) for type II errors (beta), we estimated that at least 77 MM patients and 30 healthy volunteers should be included in the study. Specifically considering polyneuropathy and the mean IL-8 serum concentration with its standard deviation (SD) in affected patients (25598.46 ± 17367.01 fg/ml) versus unaffected patients (19335.61 ± 15016.80 fg/ml), and a ratio of 1.5 between the two groups, we estimated the study’s statistical power at 84.3% for our sample size of 81 patients. All tests were two-sided, and we considered p-values less than 0.05 as statistically significant. We applied the Bonferroni correction to adjust the significance threshold for multiple comparisons.

## 3 Results

### 3.1 Characteristics of the study group

We included 81 patients with MM (50.6% were men) in the study. The median age in the study group was 65 years (interquartile range: 56–74 years). The predominant diagnosis was the MM with monoclonal protein (87.7%). In addition, ten patients had a light chain disease. The cohort was devoid of nonsecretory disease or solitary plasmacytoma cases. Moreover, during the recruitment period, we diagnosed no patients with Immunoglobulin D (IgE) or Immunoglobulin E (IgE) myeloma. Most patients were in stage 3 of the disease, according to the Durie-Salomon classification (85.2%) and the ISS classification (43.7%). Most patients were in the ECOG stage ≤1 (50.6%). 22.2% of patients had high-risk myeloma defined by the presence of the translocations t (4, 14), t (14, 16), or the deletion 17p (del(17p) (49). No patients exhibited Double-Hit or Triple-Hit MM (the presence of any two high-risk factors and three or more high-risk factors, respectively) (40). All patients received CTH, most of them the thalidomide-based regimen (Cyclophosphamide, Thalidomide, Dexamethasone (CTD); 46,9%), 34.6% bortezomib-based regimens (Bortezomib, (Cyclophosphamide), Dexamethasone (V(C)D)/Bortezomib, Melphalan, Prednisone (VMP)/Bortezomib, Adriamycin, Dexamethasone (PAD)), and 17.3% Bortezomib, Thalidomide, Dexamethasone (VTD). Moreover, 40.2% of patients underwent high-dose CTH followed by the auto-HSCT procedure. The CTH regimens were considered a standard of care during the recruitment period, and the CTH choice was based on the patient’s clinical status and current recommendations. 74.3% of patients had pretreatment anemia - mild (29.6%), moderate (32.1%), severe (12.3%), or life-threatening (1.2%). Demographics and clinical details are further elucidated in **Table 1**.

**Table 1:** Characteristics of the study group.

Variable	Study group (n=81)
Sex	
Women	40 (49.4%)
Men	41 (50.6%)
Age [years]	
Median [interquartile range]	65 [56–74]
Min-Max	42.-87
Age	
Below 65 years	39 (48.1%)
Above 65 years	42 (51.9%)
Place of residence	
City	48 (59.3%)
Village	33 (40.7%)
Other neoplasms	
No	76 (93.8%)
Yes	5 (6.2%)
Neoplasms in the family	
No	48 (61.5%)
Yes	30 (38.5%)
Exposure to chemical or physical agents	
No	53 (67.9%)
Yes	25 (32.1%)
Smoking	
No	59 (73.7%)
Yes	9 (11.3%)
Ex-smoker	12 (15.0%)
Diagnosis	
Disease with monoclonal protein	71 (87.7%)
Light chain disease	10 (12.3%)
A type of monoclonal protein	
IgA	20 (27.8%)
IgG	52 (72.2%)
Light chains	
Kappa	48 (59.3%)
Lambda	33 (40.7%)
Durie-Salmon stage	
I	3 (3.7%)
II	9 (11.1%)
III	69 (85.2%)
ISS stage	
1	22 (27.5%)
2	23 (28.7%)
3	35 (43.7%)
Renal function A/B	
A	66 (81.5%)
B	15 (18.5%)
Weight loss	
No	41 (51.2%)
5% in 3 months	15 (18.8%)
10% in 3 months	24 (30.0%)
ECOG scale	
0	7 (8.6%)
1	34 (42.0%)
2	25 (30.9%)
3	12 (14.8%)
4	3 (3.7%)
17p deletion	
No	44 (83.0%)
Yes	9 (17.0%)
Translocation t (4;14)	
No	47 (88.7%)
Yes	6 (11.3%)
Translocation (14;16)	
No	50 (94.3%)
Yes	3 (5.7%)
CTH regimen	
CTD	38 (46.9%)
V(C)D, VMP, PAD	28 (35.7%)
VTD	14 (17.3%)
Endoxan	1 (1.2%)
Bone fractures	
No	47 (58.7%)
Yes	33 (41.2%)
Supportive treatment	
No	21 (25.9%)
Zoledronic acid	12 (14.8%)
Disodium pamidronate	48 (59.3%)
auto-HSCT	
No	45 (59.2%)
Yes	31 (40.8%)
Grade of anemia before treatment in WHO classification	
Non-anemia	20 (25.7%)
Mild	24 (29.6%)
Moderate	26 (32.1%)
Severe	10 (12.3%)
Life-threatening	1 (1.2%)

Auto-HSCT, autologous hematopoietic stem cell transplantation; C, Cyclophosphamide; CTH, Chemotherapy; D, Dexamethasone; ECOG, Eastern Cooperative Oncology Group; ISS, International Staging System; IgA, Immunoglobulin A; IgG, Immunoglobulin G; P, Prednisolone; PAD, Bortezomib, Adriamycin, Dexamethasone; T, Thalidomide; V, Bortezomib, WHO, World Health Organization.

### 3.2 Comparison of serum concentrations of tested cytokines between the study and control group

The median IL-6 in the study group was significantly higher compared to the control group (13150.2 (IRQ: 2522.3 to 56874.2) vs. (3116.0 (interquartile range (IRQ): 1793.7–4939.0) respectively; p=0.0048; **Supplementary Figure 2A**). A significantly lower median IL-8 was observed in the control group compared to the study group (4555.0 vs 14567.0, respectively; p<0.0001; **Supplementary Figure 2B**). There was a significantly lower median VEGF in the control group compared to the study group (31.1 vs. 39.5, respectively; p=0.0059; **Supplementary Figure 2C**). Conversely, the median level of MCP-1 in the control group was significantly higher than in the study group (228.3 vs. 161.6, respectively p=0.0001; **Supplementary Figure 2D**).

### 3.3 Toxicity profiles

The toxicity profile was varied, with infections (53.1%), neutropenia (58%), and anemia (82.7%) being the most prevalent. Other treatment-induced toxicities included lymphopenia (69.1%), polyneuropathy (38.8%), gastrointestinal disturbances such as diarrhea (13.6%) and constipation (31.3%), thromboembolic events (7.5%), and a spectrum of less common adverse effects (40.7%). The gradation of each toxicity type was anchored in the CTCAE v 5.0 criteria. Detailed data showing the occurrence of the tested treatment-induced toxicities are in **Table 2**.

**Table 2:** Characteristics of the study group in terms of specific treatment-induced toxicity.

Variable	Study group (n=81)
Infections	
Grade 0	38 (46.9%)
Grade 1	2 (2.5%)
Grade 2	25 (30.9%)
Grade 3	13 (16.0%)
Grade 4	3 (3.7%)
Neutropenia	
Grade 0	34 (42.0%)
Grade 1	22 (27.2%)
Grade 2	8 (9.9%)
Grade 3	15 (18.5%)
Grade 4	2 (2.5%)
Anemia	
Grade 0	14 (17.3%)
Grade 1	24 (29.6%)
Grade 2	23 (28.4%)
Grade 3	19 (23.5%)
Grade 4	1 (1.2%)
Thrombocytopenia	
Grade 0	55 (67.9%)
Grade 1	14 (17.3%)
Grade 2	10 (12.3%)
Grade 3	1 (1.2%)
Grade 4	1 (1.2%)
Lymphopenia	
Grade 0	25 (30.9%)
Grade 1	11 (13.6%)
Grade 2	15 (18.5%)
Grade 3	22 (27.2%)
Grade 4	8 (9.9%)
Polyneuropathy	
Grade 0	49 (61.2%)
Grade 1	8 (10.0%)
Grade 2	18 (22.5%)
Grade 3	5 (6.2%)
No data=1	
Diarrhea	
Grade 0	70 (86.4%)
Grade 1	2 (2.5%)
Grade 2	7 (8.6%)
Grade 3	2 (2.5%)
Constipation	
Grade 0	55 (68.7%)
Grade 1	3 (3.7%)
Grade 2	21 (26.2%)
Grade 3	1 (1.3%)
No data=1	
Thromboembolic complications	
Grade 0	74 (92.5%)
Grade 1	–
Grade 2	3 (3.7%)
Grade 3	1 (1.3%)
Grade 4	1 (1.3%)
Grade 5	1 (1.3%)
Other toxicities^*^	
No	48 (59.3%)
Yes	33 (40.7%)

^*^ Other toxicities are defined in the Materials and Methods section.

### 3.4 Comparisons of the tested cytokines concentration depending on the occurrence of specific treatment-induced toxicity

#### 3.4.1 IL-6

No significant association was discerned between IL-6 levels and the tested treatment-induced toxicities.

#### 3.4.2 IL-8

**Figure 1:**
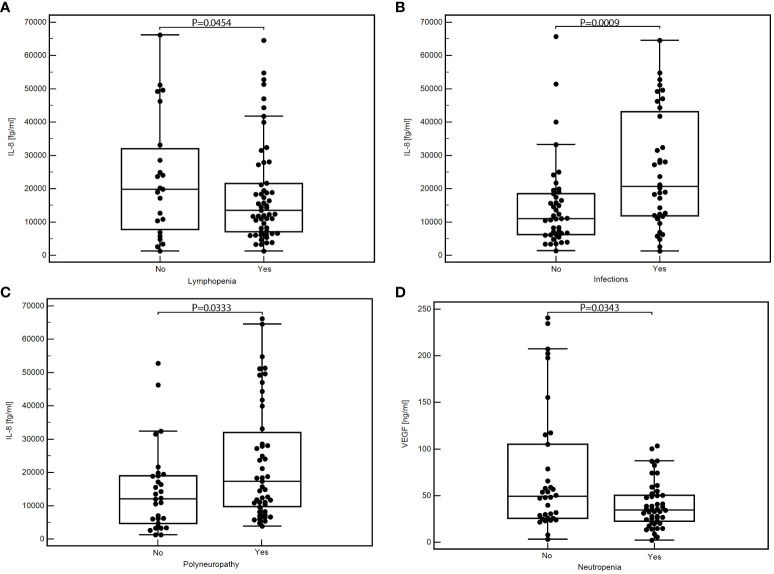
Comparison of the concentration of selected cytokines depending on the occurrence of specific toxicities of treatment: IL-8 concentration depending on the presence of lymphopenia **(A)**, infection **(B)**, or polyneuropathy **(C)**; VEGF concentration depending on the presence of neutropenia **(D)**; MedCalc 15.8 PL (MedCalc Software Ltd, Belgium). IL-6, interleukin-6; IL-8, interleukin-8; VEGF, vascular endothelial growth factor.

Patients who developed lymphopenia showed a significantly lower median concentration of IL-8 than those who did not have lymphopenia (medians 13899.46 vs. 33111.58 fg/ml; *P=*0.0454; **Figure 1A**). Conversely, patients who experienced infections presented significantly higher levels of IL-8 than those who did not (medians: 20678.10 vs. 7110.62; *P=*0.0009; **Figure 1B**). Additionally, a substantial elevation in IL-8 was observed in patients with treatment-induced polyneuropathy versus those unaffected (medians: 17768.27 vs. 12218.39 fg/ml; *P=*0.0333; **Figure 1C**).

#### 3.4.3 VEGF

We found significantly lower VEGF concentrations in patients with neutropenia than those without this toxicity (medians: 34.65 vs. 49.27 pg/ml; *P=*0.0343; **Figure 1D**). In addition, patients with other less frequent toxicities also displayed significantly reduced VEGF levels against those without such toxicities (medians: 30.43 vs. 48.94 pg/ml; *P=*0.0039; **Supplementary Figure 3**). Detailed data showing the comparison of the serum concentration of tested cytokines depending on the occurrence of specific treatment toxicity are in **Table 3**.

**Table 3:** Comparison of the serum concentration of tested cytokines depending on the occurrence of specific treatment toxicity.

Variable	Study group (n=81)	IL-6 [fg/ml]		IL-8 [fg/ml]		VEGF [pg/ml]		MCP-1 [pg/ml]	
		Median. interquartile range	*P*	Median. interquartile range	*P*	Median. interquartile range	*P*	Median. interquartile range	*P*
**Neutropenia** No Yes	34 (42.0%) 47 (58.0%)	14283.60 [3949.94–62369.06] 12543.12 [2422.8–27201.61]	0.6737	13241.44 [6141.32–44367.57] 16510.75 [9555.49–27859.05]	0.6827	49.27 [25.80–105.03] 34.65 [22.70–50.12]	0.0343*	136.19 [101.35–212.71] 174.53 [113.15–243.46]	0.1640
**Anemia** No Yes	14 (17.3%) 67 (82.7%)	9305.31 [1221.31–15071.58] 13521.01 [3968.71–62103.01]	0.2771	5950.07 [3317.62–19816.70] 16053.48 [9555.49–31515.43]	0.1369	28.11 [21.56–35.17] 40.90 [24.89–59.21]	0.2894	95.18 [66.19–161.66] 157.08 [108.41–234.23]	0.0763
**Thrombocytopenia** No Yes	55 (67.9%) 26 (32.1%)	10300.03 [1362.98–60197.20] 13773.80 [4283.85–35925.26]	0.5305	17285.32 [7292.95–23092.87] 14635.30 [7229.50–39959.37]	0.7791	34.23 [25.57–59.90] 40.23 [26.30–51.98]	0.7945	200.64 [117.73–241.15] 142.07 [83.04–202.21]	0.1004
**Lymphopenia** No Yes	25 (30.9%) 56 (69.1%)	12804.80 [1267.43–67313.06] 13019.37 [3996.08–49301.81]	0.8700	33111.58 [-] 13899.46 [7110.62–24400.68]	0.0454^*^	51.98 [-] 33.99 [24.13–51.88]	0.1572	148.97 [-] 162.65 [109.06–233.94]	0.5575
**Infections** No Yes	38 (46.9%) 43 (53.1%)	9976.35 [1282.81–20041.21] 13548.22 [4029.34–63423.35]	0.2185	7110.62 [4730.94–13534.43] 20678.10 [11975.02–44367.57]	0.0009^*^	38.30 [28.67–60.94] 32.48 [25.14–48.39]	0.2034	176.53 [111.04–298.63] 166.89 [131.16–233.65]	0.4720
**Polyneuropathy** No Yes	49 (61.2%) 31 (38.7%)	13641.99 [4094.27–62380.25] 7329.64 [1640.69–45633.76]	0.3984	12218.39 [5050.40–19298.51] 17768.27 [9952.91–36535.47]	0.0333^*^	37.14 [24.17–55.93] 41.12 [26.31–74.35]	0.3715	167.88 [111.92–233.36] 147.59 [103.07–137.83]	0.2401
**Diarrhea** No Yes	70 (86.4%) 11 (13.6%)	11421.57 [2295.82–56874.22] 13654.58 [5062.09–43689.98]	0.7200	14496.47 [7170.06–25436.38] 17285.32 [7958.68–45723.76]	0.3980	37.84 [25.41–58.84] 38.77 [24.21–69.41]	0.8669	163.65 [107.03–243.63] 138.28 [95.23–220.00]	0.3980
**Constipation** No Yes	55 (68.7%) 25 (31.2%)	12804.80 [2352.44–43689.98] 13521.01 [3559.89–43689.98]	0.7912	15185.11 [6870.38–28088.02] 16370.78 [9205.26–28034.96]	0.9244	35.12 [25.14–58.72] 47.25 [22.59–67.72]	0.6466	164.38 [106.91–239.23] 150.36 [105.64–245.11]	0.7043
**Thromboembolic complications** No Yes	74 (92.5%) 6 (7.5%)	12673.96 [2389.75–46278.22] 21627.45 [4321.01–87652.11]	0.6348	14823.57 [6795.03–27916] 22521.10 [10889.48–41758.89]	0.3267	38.77 [24.92–58.84] 45.51 [24.89–74.28]	0.8823	157.09 [107.03–235.48] 176.28 [90.26–271.28]	0.8823
Other toxicities^**^ No Yes	48 (59.3%) 33 (40.7%)	13508.31 [1859.07–59621.64] 12543.12 [4006.27–38513.50]	0.8550	15958.71 [6704.58–25626.04] 15736.18 [7923.31–44845.57]	0.6376	48.94 [30.50–84.64] 30.43 [19.17–49.96]	0.0039^*^	164.38 [115.04–236.73] 150.36 [96.11–234.47]	0.6615

*Statistically significant result.

^**^ Other toxicities are defined in the Materials and Methods section.

CTCAE, Common Terminology Criteria for Adverse Events, IL-6, interleukin-6; IL-8, interleukin-8; MCP-1, angiogenic chemokine monocyte chemoattractant protein-1; VEGF, vascular endothelial growth factor.

### 3.5 Relationship between the level of cytokines tested and the risk of specific treatment toxicity

In univariate analyses, elevated IL-8 levels were significantly linked to a reduced risk of lymphopenia (Odds Ratio [OR] = 0.33; *P*=0.0320), while being associated with a markedly increased risk of infections (OR = 4.54, *P*=0.0028) and polyneuropathy (OR = 3.84, P=0.0212). Multivariate analyses further established IL-8 as an independent predictor for both lymphopenia (OR = 0.26; 95% Confidence Interval [CI] = 0.07–0.78; *P*=0.0167) and infection (OR = 4.76; 95% CI = 0.07–0.62; *P*=0.0049).

Similarly, in univariate analysis, elevated VEGF levels correlated with a quadrupled risk of anemia (OR = 4.13; P=0.0414), although multivariate analysis did not demonstrate significant risk variations for the toxicities tested relative to VEGF and MCP-1 levels.

For comprehensive insights into the data, please refer to **Supplementary Tables 1** and **2**, which detail the relationships between cytokine levels and the risk of specific treatment-related toxicities.

### 3.6 Correlations between the concentrations of the cytokines tested and the degrees of toxicity associated with the treatment

**Figure 2:**
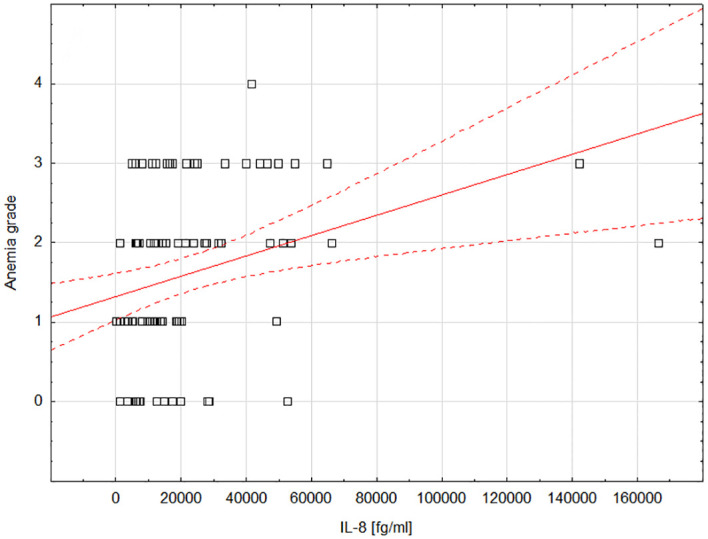
Scatterplot showing the correlation between the degree of anemia and the concentration of IL-8; Statistica 13 PL (StatSoft, USA). IL-8, interleukin-8.

We observed a statistically significant positive correlation between IL-8 and anemia grade during treatment (moderate correlation; rho=0.372; *P=*0.0007; **Figure 2**). **Supplementary Table 3** includes detailed data showing the correlations between the tested cytokines and treatment-induced toxicity.

### 3.7 Diagnostic value of cytokine concentrations for treatment-induced toxicity

**Supplementary Tables 4–6** offer a comprehensive overview of the diagnostic accuracy of IL-6, IL-8, and VEGF, respectively, in the context of treatment-induced toxicities.

#### 3.7.1 IL-8

**Figure 3:**
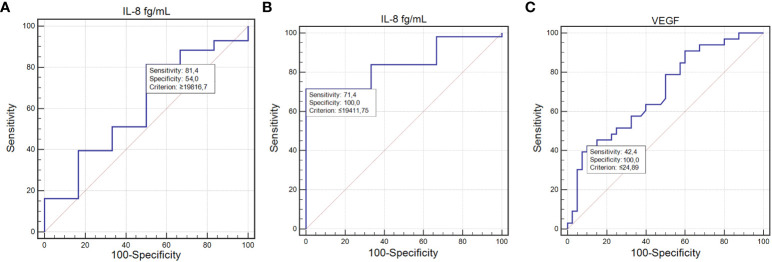
ROC curve illustrating the diagnostic usefulness of the concentration of tested cytokines in detecting toxicities occurring during treatment: IL-8 concentration in the detection of infections **(A)**; IL-8 concentration in the detection of lymphopenia **(B)**; VEGF concentration in the detection of other toxicities **(C)** (MedCalc 15.8 PL (MedCalc Software Ltd, Belgium). IL-6, interleukin-6; IL-8, interleukin-8.

IL-8’s diagnostic performance was notable, with a sensitivity of 81% and specificity of 54% for detecting infections (AUC=0.69; P=0.0015; **Figure 3A**). For lymphopenia, IL-8 achieved a sensitivity of 71% and specificity of 100%, indicating a strong diagnostic potential (AUC=0.84; P=0.0001; **Figure 3B**).

#### 3.7.2 VEGF

The ability of VEGF to predict other, less frequent toxicities was marked by a sensitivity of 42.42% and a specificity of 100%, with an AUC of 0.69 (P=0.0014; **Figure 3C**).

## 4 Discussion

The tumor microenvironment (TME) in (MM) is intricately shaped by cytokines and soluble mediators, which play crucial roles in its dynamics. Our study highlights the significant prognostic and predictive value of cytokines such as IL-6, IL-8, and VEGF, underscoring their potential as biomarkers for chemotherapy toxicity and their influence on TME reprogramming.

We discovered a notable lack of research on the predictive value of pretreatment cytokine levels for chemotherapy-induced toxicity in MM in the available databases (MEDLINE and Cochrane). Thus, we aimed to address this knowledge gap.

In our study, elevated IL-8 levels were linked to a nearly 4.5-fold increase in infection risk, establishing it as an independent predictor of infection. The role of IL-8 in systemic inflammatory responses remains little understood (50–52). It is known to play key roles in neutrophil chemotaxis, lysosomal enzyme release, adhesion molecule upregulation, intracellular calcium increase, and oxidative burst initiation (53, 54). We did not identify studies specifically evaluating pretreatment IL-8 levels and their association with infection rates during CTH for MM. Wu et al. demonstrated the clinical utility of IL-8, alongside interleukin-2 receptor (IL-2R), in identifying febrile infections in patients with hematologic malignancies (55). This is consistent with van de Geer et al., who also underscored the diagnostic importance of IL-8 in similar settings (56). Additionally, Angel et al. proposed that IL-8 levels at the onset of fever might indicate serious complications like bacteremia, aiding in the early identification of patients needing immediate intensive care (57). These insights are particularly relevant for MM, where infections remain a primary cause of mortality in the early stages post-diagnosis (58, 59). Thus, the predictive application of IL-8 levels could enhance patient monitoring, potentially leading to swifter therapeutic interventions.

Our study offers new insights, showing that lower pre-treatment IL-8 levels significantly correlate with treatment-induced lymphopenia. Interestingly, IL-8 demonstrated specificity and sensitivity as a marker in our multivariate analysis. This contrasts with findings by Chung et al., where elevated IL-8 levels were observed in patients with septic shock and severe lymphopenia (60). The discrepancy likely stems from the inflammatory milieu associated with sepsis, which acutely increases IL-8, a pro-inflammatory cytokine, unlike the stable pre-chemotherapy conditions of our MM patient cohort.

Our study highlighted complex relationships where increased IL-8 levels prior to treatment were linked to a lower risk of lymphopenia but a higher occurrence of infectious complications. This underscores the intricate dynamics between IL-8 concentrations and treatment outcomes. Importantly, serum chemokine and cytokine levels may not fully capture the inflammatory response, as these mediators often operate locally through autocrine signaling. The complexity of the inflammatory response, influenced by a wide range of pathogens, including viruses, bacteria, and fungi, further complicates our findings. Our analysis did not consider the specific etiology of the infections, a factor that could have enriched our understanding of the observed phenomena. Our study demonstrated a significant positive correlation between IL-8 levels and anemia severity during chemotherapy. Reports suggest that IL-8 can inhibit myelopoiesis by suppressing the proliferation of bone marrow progenitor cells through signaling inhibition via its receptor ligand (28). This mechanism highlights the complex role of IL-8 in inflammatory responses and in the regulation of hematopoiesis.

While there is limited research directly linking pretreatment IL-8 levels to anemia during chemotherapy, previous studies have shown a connection between high IL-8 levels and severe aplastic anemia, marked by bone marrow failure (61, 62). Moreover, our analysis identified high VEGF levels as a potential risk factor, with elevated concentrations conferring a roughly fourfold increased risk of developing anemia. This aligns with studies suggesting anemia-induced hypoxia may trigger VEGF secretion, fostering angiogenesis as an adaptive response (63, 64). Considering the elevated VEGF levels observed in more advanced MM cases (30), it is plausible to speculate a correlation where a greater tumor burden could exacerbate bone marrow suppression, thus impeding hemoglobin synthesis during treatment.

Our findings highlight that high IL-8 levels in patients experiencing chemotherapy-induced polyneuropathy correlate with nearly a fourfold increase in risk. Considering polyneuropathy’s common occurrence, especially in patients treated with bortezomib or thalidomide for MM (65), the prognostic value of IL-8 could be pivotal. Our data suggest IL-8’s potential as a biomarker to identify patients at heightened risk of neurotoxicity, offering a pathway toward more personalized treatment strategies.

IL-8 can be produced by various cell types involved in inflammation, including monocytes and endothelial cells. Activation of these cells occurs upon binding with IL-8 receptors, CXCR1 and CXCR2, which are expressed on neutrophils, monocytes, endothelial cells, astrocytes, and microglia (66). IL-8 may significantly influence inflammatory cell mediation in acute and chronic inflammation phases (67).

Studies have linked elevated pro-inflammatory cytokines, including IL-8, to neuropathy in patients with conditions like POEMS syndrome related to osteosclerotic myeloma and neuropathy (68).

Moreover, our findings reveal that lower pre-chemotherapy serum VEGF levels are significantly associated with the onset of treatment-induced neutropenia, indicating a predictive role for VEGF. Further validation of this biomarker could lead to preemptive adjustments in treatment strategies for cancer patients. Neutropenia is a significant challenge in clinical oncology, greatly increasing the risk of severe infections that may necessitate hospitalization, intensive antibiotic treatments, and potentially disrupt crucial chemotherapy regimens (69–73). The economic burden is also notable, as neutropenia-associated hospitalization costs exceeded $2 billion in the U.S. by 2012 (74).

To date, the correlation between MCP-1 levels at diagnosis and subsequent treatment-related toxicities remains uncharted, presenting a novel focus for our pilot study. Valković et al. previously established a link between elevated MCP-1 levels and the primary clinical manifestations of MM, finding that patients with increased pretreatment MCP-1 concentrations experienced heightened severity of bone disease, renal dysfunction, and anemia (75). They posited that this correlation could be partly attributed to reduced renal clearance of MCP-1 due to renal insufficiency, warranting a cautious interpretation of these findings.

Contrary to these associations, our investigation did not observe a statistically significant role for MCP-1 concerning treatment toxicities in MM patients. This could indicate that the influence of MCP-1 may be more nuanced or that our study’s scale was insufficient to detect such associations.

While our study did not focus on the toxicity profiles of individual CTH regimens, our group’s toxicity profile aligns well with findings from previous research. For example, the MRC Myeloma IX trial, which evaluated the CTD regimen for newly diagnosed multiple myeloma (NDMM) patients, reported similar rates of cytopenia (15.5%), sensory neuropathy (23.7%), motor neuropathy (11.7%), constipation (41%), and infection (32.1%) to those observed in our study (76).

CASSIOPEIA study (Bortezomib, thalidomide, and dexamethasone with or without daratumumab before and after autologous stem-cell transplantation for NDMM) used VTD chemotherapy regimen as a control arm (77). Detailed safety analysis showed higher levels of peripheral sensory neuropathy (63%) than in our study (39%). This difference might stem from the greater neurotoxicity of the VTD regimen, which was administered to only 17% of our patients. On the other hand, the study reported comparable levels of hematological toxicities that were observed in our cohort. The meta-analysis of VCD vs. VTD-based regimens as induction therapies in NDMM patients eligible for transplantation showed that peripheral neuropathy grade 3 or higher was 6%, which directly aligned with the results of our study (78).

## 5 Limitations

The cohort size, while aligned with global median age, gender distribution, and disease type for MM, was relatively small, limiting the generalizability of our results. Our selection criteria aimed to create a homogeneous patient group to minimize confounding variables, excluding patients with autoimmune diseases due to their distinct cytokine profiles. This selective demographic may limit the applicability of our findings to a broader MM population. Our methodology focused primarily on the diagnostic performance of cytokine levels using standard statistical tools like ROC analysis. This analysis did not incorporate functional assessments of cytokines, which could provide deeper insights into their roles in MM pathogenesis. Moreover, it did not consider changes in cytokine levels over time or in response to treatment, which are important dynamics that could affect their diagnostic value.

Another limitation is our exclusive focus on serum cytokine levels, neglecting the potential insights that could be gained from analyzing bone marrow samples.

Additionally, our study did not measure cytokine levels in patients actively undergoing chemotherapy, nor did it consider the timing of these measurements in relation to subsequent toxicity assessments. The diversity of chemotherapy regimens within our study cohort, while reflective of real-world clinical settings, resulted in subgroups that were too small to determine statistical significance regarding the impact of treatment choice on cytokine levels and toxicity risks. Furthermore, we collected extensive data on comorbidities and concurrent medications but chose not to include these in our primary analysis. This decision was based on preliminary findings suggesting minimal impact of these variables.

Despite these limitations, our research provides important insights into the predictive value of cytokine concentrations for managing chemotherapy-induced toxicities in MM, paving the way for future studies aimed at refining predictive models for treatment complications and ultimately enhancing the development of personalized treatment approaches (79, 80).

## 6 Conclusions

Our research presents compelling evidence that assessing cytokine concentrations, specifically IL-8 and VEGF, can be useful in predicting hematological complications such as lymphopenia, neutropenia, anemia, infection, and polyneuropathy in MM patients.

The potential of these cytokines to predict chemotherapy toxicity, while promising, should be approached with prudence.

This need for cautious optimism underscores the preliminary nature of our results and the imperative for additional studies. A larger, more diverse patient population would enable a more robust analysis, affirming or refuting the capacity of these cytokines as reliable predictive biomarkers in MM therapy.

In summary, while our study contributes to the evolving narrative of cytokine profiles in MM and their association with treatment response, it also highlights the complexity of translating these findings into clinical practice. By deepening our understanding and continuing to investigate these potential biomarkers, we move closer to realizing the promise of personalized medicine in MM treatment, where every patient’s therapeutic pathway is tailored to their unique cytokine landscape.

## Data availability statement

The raw data supporting the conclusions of this article will be made available by the authors, without undue reservation.

## Ethics statement

Each patient gave written informed consent for the study. The Committee of Ethics and Research approved the study design at the Medical University of Lublin (consent no.: KE-0254/26/2015 with its actualization KE-0254/77/2019).

## Author contributions

MM: Conceptualization, Formal Analysis, Investigation, Methodology, Writing – original draft, Writing – review & editing, Validation. MP-M: Investigation, Methodology, Validation, Writing – original draft, Writing – review & editing. AS-S: Conceptualization, Data curation, Formal Analysis, Funding acquisition, Investigation, Methodology, Project administration, Resources, Supervision, Validation, Writing – original draft, Writing – review & editing. IH-M: Data curation, Formal Analysis, Methodology, Software, Validation, Visualization, Writing – original draft, Writing – review & editing. RM: Data curation, Formal Analysis, Methodology, Software, Validation, Visualization, Writing – original draft, Writing – review & editing. AG: Data curation, Software, Writing – original draft. MH: Funding acquisition, Investigation, Resources, Supervision, Validation, Writing – review & editing.
