# Supplementary material for: Predicting chemotherapy toxicity in multiple myeloma: the prognostic value of pre-treatment serum cytokine levels of interleukin-6, interleukin-8, monocyte chemoattractant protein-1, and vascular endothelial growth factor
Source: Front Immunol. 2024 May 23;15:1377546. doi: 10.3389/fimmu.2024.1377546 (PMC11153710; doi:10.3389/fimmu.2024.1377546)
Supplement: Supplementary file 1 [file Table_1.docx]

Supplementary Material

# Supplementary Tables

**Supplementary Table 1**. Relationship between IL-6 and IL-8 levels and the risk of specific treatment toxicities.

| **Variable** | **Study group**  **(n=81)** | **IL-6 [fg/ml]** | | | | **IL-8 [fg/ml]** | | | |
| --- | --- | --- | --- | --- | --- | --- | --- | --- | --- |
|  |  | **Univariate analysis** | | **Multivariate analysis** | | **Univariate analysis** | | **Multivariate analysis** | |
|  |  | **OR 95%CI** | ***p*** | **OR** | ***p*** | **OR 95%CI** | ***p*** | **OR**  **95%CI** | ***p*** |
| **Neutropenia**  No  Yes | 34 (42.0%)  47 (58.0%) | 0.78  [0.32- 1.89] | 0.5861 | 1.40  [0.53-3.74] | 0.4915 | 1.50  [0.61-3.67] | 0.3665 | 1.50  [0.53-4.22] | 0.4408 |
| **Anemia**  No  Yes | 14 (17.3%)  67 (82.7%) | 1.96  [0.59-6.49] | 0.2660 | 1.54  [0.58-4.05] | 0.3755 | 2.03  [0.61-6.71] | 0.2449 | 0.94  [0.22-3.95] | 0.9425 |
| **Thrombocytopenia**  No  Yes | 55 (67.9%)  26 (32.1%) | 1.63  [0.63-4.19] | 0.3054 | 0.42  [0.15-1.19] | 0.1059 | 1.00  [0.39-2.54] | 1.0000 | 1.18  [0.39-3.14] | 0.8317 |
| **Lymphopenia**  No  Yes | 25 (30.9%)  56 (69.1%) | 1.08  [0.42-2.78] | 0.8679 | 0.58  [0.21-1.60] | 0.2982 | 0.33  [0.12-0.91] | 0.0320* | 0.26  [0.08-0.78] | 0.0167* |
| **Infections**  No  Yes | 38 (46.9%)  43 (53.1%) | 1.42  [0.59-3.41] | 0.4324 | 0.54  [0.21-1.40] | 0.2100 | 4.54  [0.08-0.59] | 0.0028* | 4.76  [0.07-0.62] | 0.0049* |
| **Polyneuropathy**  No  Yes | 49 (61.2%)  31 (38.7%) | 0.58  [0.23-1.46] | 0.2528 | 0.68  [0.25-1.83] | 0.4515 | 3.84  [0.08-0.82] | 0.0212* | 2.32  [0.14-1.33] | 0.1455 |
| **Diarrhea**  No  Yes | 70 (86.4%)  11 (13.6%) | 1.27  [0.35-4.55] | 0.7130 | 0.74  [0.19-2.82] | 0.6634 | 1.90  [0.51-7.11] | 0.3356 | 1.14  [0.29-4.49] | 0.8459 |
| **Constipation**  No  Yes | 55 (68.7%)  25 (31.2%) | 1.12  [0.43-2.89] | 0.8094 | 0.82  [0.30-2.26] | 0.7084 | 1.08  [0.41-2.79] | 0.8687 | 0.54  [0.18-1.61] | 0.2724 |
| **Thromboembolic complications**  No  Yes | 74 (92.5%)  6 (7.5%) | 1.05  [0.19-5.57] | 0.9492 | 0.42  [0.06-2.97] | 0.3923 | 2.05  [0.35-11.92] | 0.4219 | 2.30  [0.32-16.42] | 0.4056 |
| **Other toxicities**†  No  Yes | 48 (59.3%)  33 (40.7%) | 0.76  [0.31-1.86] | 0.5580 | 1.42  [0.50-3.97] | 0.5033 | 1.10  [0.45-2.70] | 0.8204 | 0.71  [0.24-2.13] | 0.5504 |

^*^ Statistically significant result

† Other toxicities are defined in the Materials and Methods section

Abbreviations: IL-6, interleukin-6; IL-8, interleukin-8

**Supplementary Table 2**. Relationship between VEGF and MCP-1 levels and the risk of specific treatment toxicity.

| **Variable** | **Study group**  **(n=81)** | **VEGF [fg/ml]** | | | | **MCP-1 [fg/ml]** | | | |
| --- | --- | --- | --- | --- | --- | --- | --- | --- | --- |
|  |  | **Univariate analysis** | | **Multivariate analysis** | | **Univariate analysis** | | **Multivariate analysis** | |
|  |  | **OR 95%CI** | ***p*** | **OR 95%CI** | ***p*** | **OR 95%CI** | ***p*** | **OR**  **95%CI** | ***p*** |
| **Neutropenia**  No  Yes | 34 (42.0%)  47 (58.0%) | 2.07  [0.84-5.08] | 0.1105 | 1.90  [-] | 0.9946 | 2.10  [0.84-5.18] | 0.1079 | 1.89  [0.72-4.96] | 0.1917 |
| **Anemia**  No  Yes | 14 (17.3%)  67 (82.7%) | 4.13  [1.05-16.21] | 0.0414* | 0.21  [0.03-1.21] | 0.0817 | 0.94  [0.29-2.98] | 0.9180 | 0.91  [0.26-3.23] | 0.8949 |
| **Thrombocytopenia**  No  Yes | 55 (67.9%)  26 (32.1%) | 1.45  [0.57-3.37] | 0.4312 | 0  [-] | 0.9936 | 0.53  [0.20-1.39] | 0.2040 | 0.55  [0.20-1.49] | 0.2425 |
| **Lymphopenia**  No  Yes | 25 (30.9%)  56 (69.1%) | 0.53  [0.20-1.41] | 0.2067 | 0.62  [0.21-1.77] | 0.3788 | 1.18  [0.45-3.08] | 0.7327 | 1.29  [0.48-3.44] | 0.6109 |
| **Infections**  No  Yes | 38 (46.9%)  43 (53.1%) | 1.12  [0.46-2.70] | 0.7963 | 1.12  [0.26-4.77] | 0.8739 | 0.54  [0.22-1.33] | 0.1855 | 0.49  [0.19-1.27] | 0.1441 |
| **Polyneuropathy**  No  Yes | 49 (61.2%)  31 (38.7%) | 1.26  [0.51-3.11] | 0.6159 | 1.55  [0.35-6.70] | 0.5566 | 0.66  [0.26-1.65] | 0.3790 | 0.66  [0.24-1.78] | 0.4200 |
| **Diarrhea**  No  Yes | 70 (86.4%)  11 (13.6%) | 0.90  [0.25-3.26] | 0.8837 | 0.67  [0.07-6.39] | 0.7340 | 0.55  [0.14-2.06] | 0.3807 | 0.55  [0.14-2.18] | 0.4005 |
| **Constipation**  No  Yes | 55 (68.7%)  25 (31.2%) | 1.59  [0.61-4.13] | 0.3406 | 1.00  [0.99-1.01] | 0.5649 | 0.72  [0.28-1.89] | 0.5168 | 0.66  [0.24-1.78] | 0.4135 |
| **Thromboembolic complications**  No  Yes | 74 (92.5%)  6 (7.5%) | 1.08  [0.20-5.73] | 0.9229 | 0.94  [0.14-6.09] | 0.9562 | 1.08  [0.20-5.73] | 0.9229 | 0.96  [0.15-5.89] | 0.9710 |
| **Other toxicities**†  No  Yes | 48 (59.3%)  33 (40.7%) | 0.57  [0.23-1.41] | 0.2255 | 0.34  [0.11-1.01] | 0.0534 | 0.79  [0.32-1.94] | 0.6214 | 0.90  [0.33-2.47] | 0.8531 |

^*^ Statistically significant result

† Other toxicities are defined in the Materials and Methods section

Abbreviations: MCP-1, angiogenic chemokine monocyte chemoattractant protein-1; VEGF, vascular endothelial growth factor

**Supplementary Table 3**. Spearman rank correlations between the serum concentrations of the tested cytokines and the degree of specific treatment-induced toxicity.

| **Variable** | **n** | **IL-6 [fg/ml]** | | **IL-8 [fg/ml]** | | **VEGF [pg/ml]** | | **MCP-1 [pg/ml]** | |
| --- | --- | --- | --- | --- | --- | --- | --- | --- | --- |
|  |  | **rho** | ***P*** | **rho** | ***P*** | **rho** | ***P*** | **rho** | ***p*** |
| **Neutropenia** | 80 | -0.047 | 0.6764 | 0.025 | 0.8227 | -0.192 | 0.0880 | 0.172 | 0.1276 |
| **Anemia** | 72 | 0.121 | 0.2799 | 0.372 | 0.0007^*^ | 0.219 | 0.0645 | 0.003 | 0.9770 |
| **Thrombocytopenia** | 49 | 0.070 | 0.5339 | 0.068 | 0.6440 | 0.083 | 0.5695 | -0.112 | 0.4444 |
| **Lymphopenia** | 59 | 0.018 | 0.8713 | -0.018 | 0.8905 | -0.270 | 0.0155 | -0.053 | 0.6914 |
| **Infections** | 75 | 0.138 | 0.2208 | 0.196 | 0.0917 | -0.078 | 0.5071 | -0.174 | 0.1356 |
| **Polyneuropathy** | 79 | -0.095 | 0.4019 | 0.209 | 0.0642 | 0.076 | 0.5052 | -0.123 | 0.2815 |
| **Diarrhea** | 80 | 0.040 | 0.7224 | 0.093 | 0.4097 | -0.027 | 0.8090 | -0.087 | 0.4406 |
| **Constipation** | 79 | 0.030 | 0.7931 | -0.008 | 0.9446 | 0.069 | 0.5473 | -0.059 | 0.6075 |
| **Thromboembolic complications** | 79 | 0.053 | 0.6378 | 0.113 | 0.3203 | 0.022 | 0.8503 | 0.020 | 0.8638 |

^*^ Statistically significant result

Abbreviations: IL-6, interleukin-6; IL-8, interleukin-8; MCP-1, angiogenic chemokine monocyte chemoattractant protein-1; VEGF, vascular endothelial growth factor

**Supplementary Table 4**. Evaluation of the usefulness of IL-6 concentration in detecting specific treatment-induced toxicity.

| **IL-6** | | | | | |
| --- | --- | --- | --- | --- | --- |
| **Variable** | **AUC**  **[95% CI]** | **Sensitivity %** | **Specificity %** | **Cut-off point** | ***P*** |
| **Infections**  No  Yes | 0.58  [0.46-0.68] | 48.84 | 71.05 | >15514.61 | 0.2177 |
| **Neutropenia**  No  Yes | 0.52  [0.41-0.64] | 76.60 | 38.24 | ≤27749.21 | 0.6805 |
| **Anemia**  No  Yes | 0.59  [0.47-0.70] | 41.79 | 85.71 | >17325.26 | 0.2587 |
| **Thrombocytopenia**  No  Yes | 0.61  [0.46-0.75] | 61.54 | 65.22 | ≤0.1 | 0.1361 |
| **Lymphopenia**  No  Yes | 0.51  [0.39-0.62] | 85.71 | 28.00 | >1282.81 | 0.8791 |
| **Polyneuropathy**  No  Yes | 0.55  [0.44-0.66] | 51.61 | 65.31 | ≤7329.64 | 0.4022 |
| **Diarrhea**  No  Yes | 0.53  [0.42-0.64] | 90.91 | 25.71 | >2295.82 | 0.6991 |
| **Constipation**  No  Yes | 0.51  [0.40-0.63] | 48.00 | 67.27 | >17325.26 | 0.7989 |
| **Thromboembolic complications**  No  Yes | 0.55  [0.44-0.67] | 50.00 | 72.97 | >27749.21 | 0.6262 |
| **Other toxicities**†  No  Yes | 0.51  [0.39-0.62] | 90.91 | 20.83 | ≤87652.11 | 0.8545 |

^*^ Statistically significant result

† Other toxicities are defined in the Materials and Methods section

Abbreviations: AUC, Area Under the Curve, CI, confidence interval, IL-6, interleukin-6

**Supplementary Table 5**. Evaluation of the usefulness of the concentration of IL-8 in detecting specific treatment-induced toxicity.

| **IL-8** | | | | | |
| --- | --- | --- | --- | --- | --- |
| **Variable** | **AUC**  **[95% CI]** | **Sensitivity %** | **Specificity %** | **Cut-off point** | ***P*** |
| **Infections**  No  Yes | 0.69  [0.58-0.79] | 81.40 | 54.05 | ≥19816.7 | 0.0015^*^ |
| **Neutropenia**  No  Yes | 0.52  [0.41-0.64] | 0.00 | 79.41 | >51165.99 | 0.7020 |
| **Anemia**  No  Yes | 0.68  [0.56-0.78] | 84.85 | 66.67 | >6141.32 | 0.2284 |
| **Thrombocytopenia**  No  Yes | 0.52  [0.37-0.66] | 30.77 | 86.96 | >32347.07 | 0.7838 |
| **Lymphopenia**  No  Yes | 0.84  [0.72-0.92] | 71.43 | 100.00 | ≤19411.75 | 0.0001^*^ |
| **Polyneuropathy**  No  Yes | 0.64  [0.52-0.74] | 83.87 | 41.67 | ≥21609.45 | 0.0290 |
| **Diarrhea**  No  Yes | 0.58  [0.46-0.68] | 45.45 | 81.16 | >28561.81 | 0.4546 |
| **Constipation**  No  Yes | 0.50  [0.39-0.62] | 68.00 | 42.59 | ≤18836.52 | 0.9250 |
| **Thromboembolic complications**  No  Yes | 0.62  [0.50-0.72] | 66.67 | 67.12 | >19816.7 | 0.2652 |
| **Other toxicities**†  No  Yes | 0.53  [0.41-0.65] | 33.33 | 90.00 | >33111.58 | 0.6431 |

^*^ Statistically significant result

† Other toxicities are defined in the Materials and Methods section

Abbreviations: AUC, Area Under the Curve, CI, confidence interval, IL-8, interleukin-8.

**Supplementary Table 6.** Evaluation of the usefulness of VEGF concentrations in detecting specific treatment-induced toxicity.

| **VEGF** | | | | | |
| --- | --- | --- | --- | --- | --- |
| **Variable** | **AUC**  **[95% CI]** | **Sensitivity %** | **Specificity %** | **Cut-off point** | ***P*** |
| **Infections**  No  Yes | 0.51  [0.36-0.65] | 41.86 | 100.00 | ≤26.3 | 0.8959 |
| **Neutropenia**  No  Yes | 0.63  [0.52-0.74] | 100 | 26.47 | ≤103.41 | 0.0317 |
| **Anemia**  No  Yes | 0.63  [0.50-0.74] | 57.58 | 83.33 | >35.17 | 0.3531 |
| **Thrombocytopenia**  No  Yes | 0.52  [0.37-0.66] | 100.00 | 17.39 | ≤87.05 | 0.8010 |
| **Lymphopenia**  No  Yes | 0.74  [0.61-0.84] | 50.00 | 100.00 | ≤32.91 | 0.0914 |
| **Polyneuropathy**  No  Yes | 0.56  [0.44-0.67] | 41.94 | 72.92 | >51.98 | 0.1485 |
| **Diarrhea**  No  Yes | 0.51  [0.40-0.62] | 36.36 | 76.81 | ≤24.89 | 0.8761 |
| **Constipation**  No  Yes | 0.53  [0.41-0.64] | 48.00 | 70.37 | >50.12 | 0.6618 |
| **Thromboembolic complications**  No  Yes | 0.51  [0.40-0.63] | 50.00 | 72.60 | >54.83 | 0.8955 |
| **Other toxicities**†  No  Yes | 0.69  [0.57-0.80] | 42.42 | 90.00 | ≤24.89 | 0.0014^*^ |

^*^ Statistically significant result

† Other toxicities are defined in the Materials and Methods section

Abbreviations: AUC, Area Under the Curve; VEGF, vascular endothelial growth factor

# Supplementary Figures


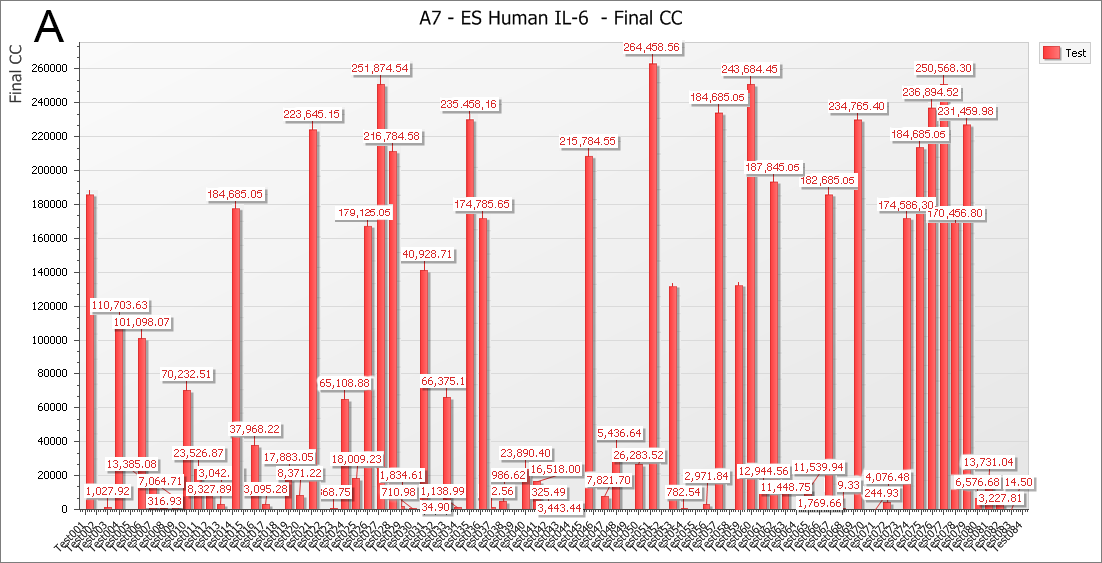

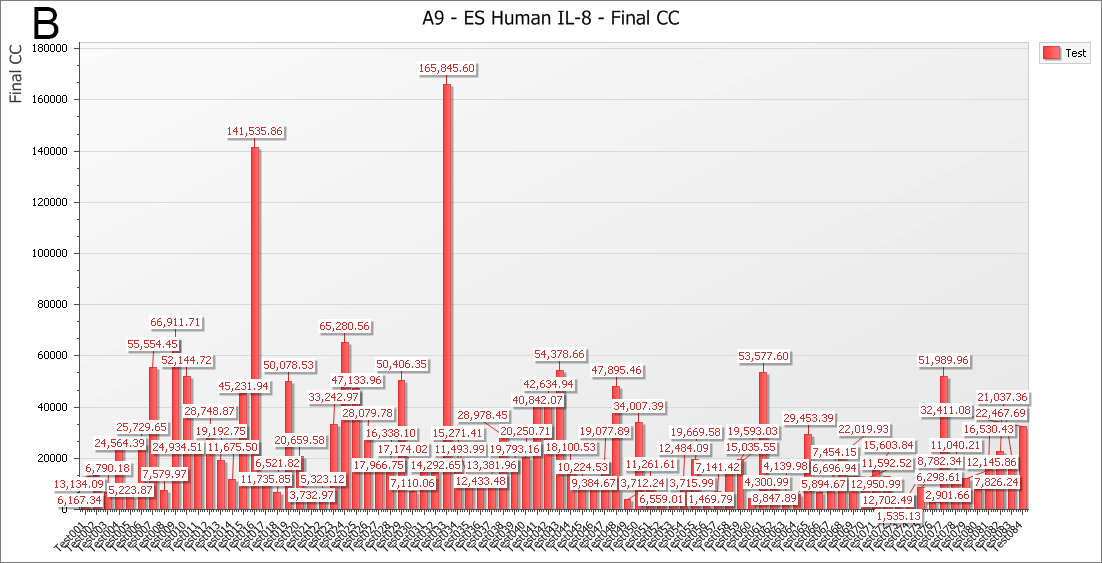


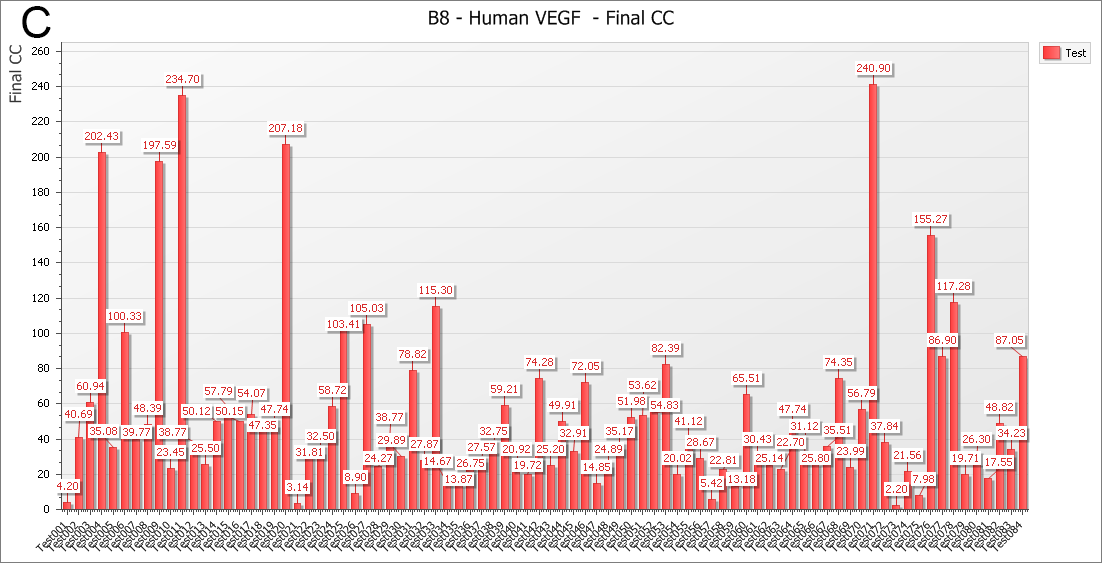

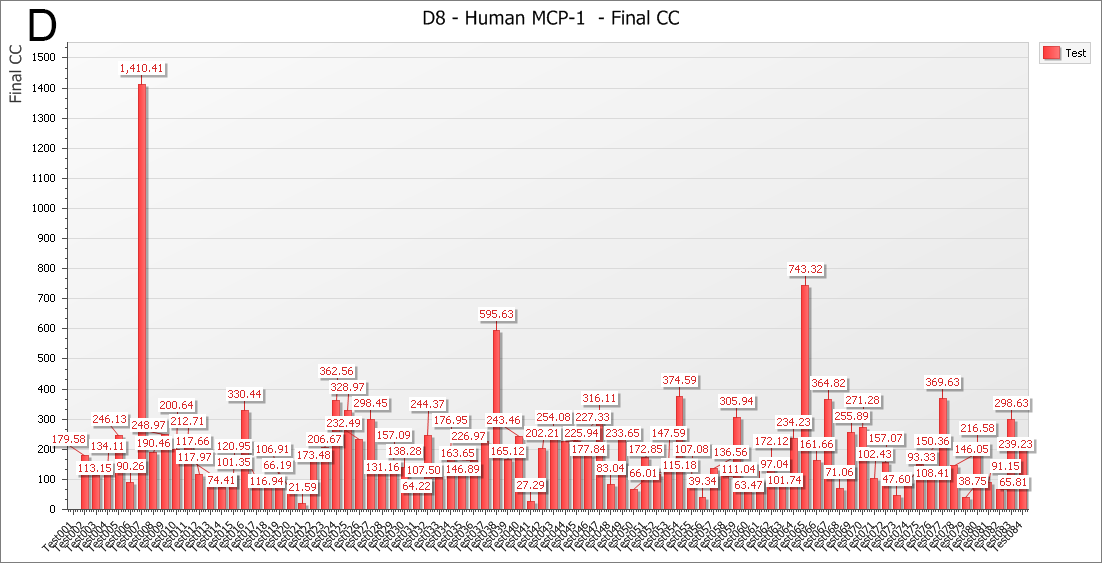


Abbreviations: IL-6, interleukin-6; IL-8, interleukin-8; VEGF, vascular endothelial growth factor; MCP-1, angiogenic chemokine monocyte chemoattractant protein-1

**Supplementary Figure 1**. Concentrations of IL-6 (A), IL-8 (B), VEGF (C), MCP-1 (D), FCAP Array™ software (Becton Dickinson, USA.


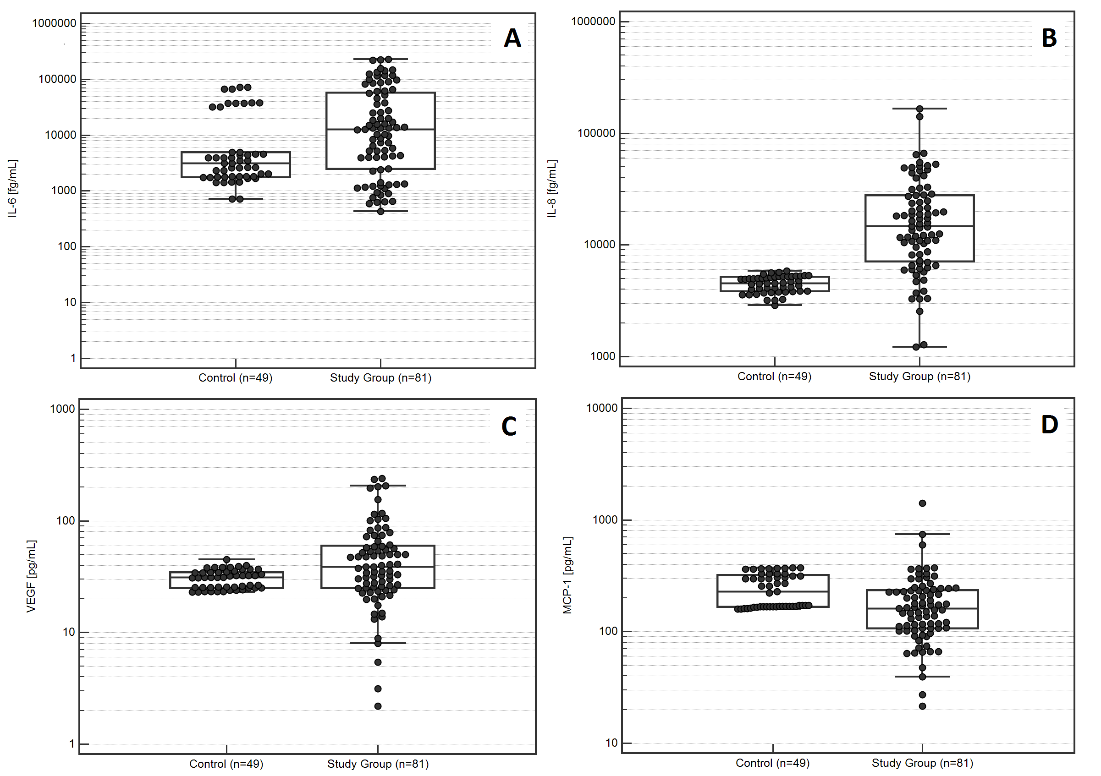


Abbreviations: IL-6, interleukin-6; IL-8, interleukin-8; VEGF, vascular endothelial growth factor; MCP-1, angiogenic chemokine monocyte chemoattractant protein-1

**Supplementary Figure 2.** Box and whisker plot comparing the concentrations of IL-6 (A), IL-8 (B), VEGF (C), MCP-1 (D) between study and control groups; MedCalc 15.8 PL (MedCalc Software Ltd, Belgium)


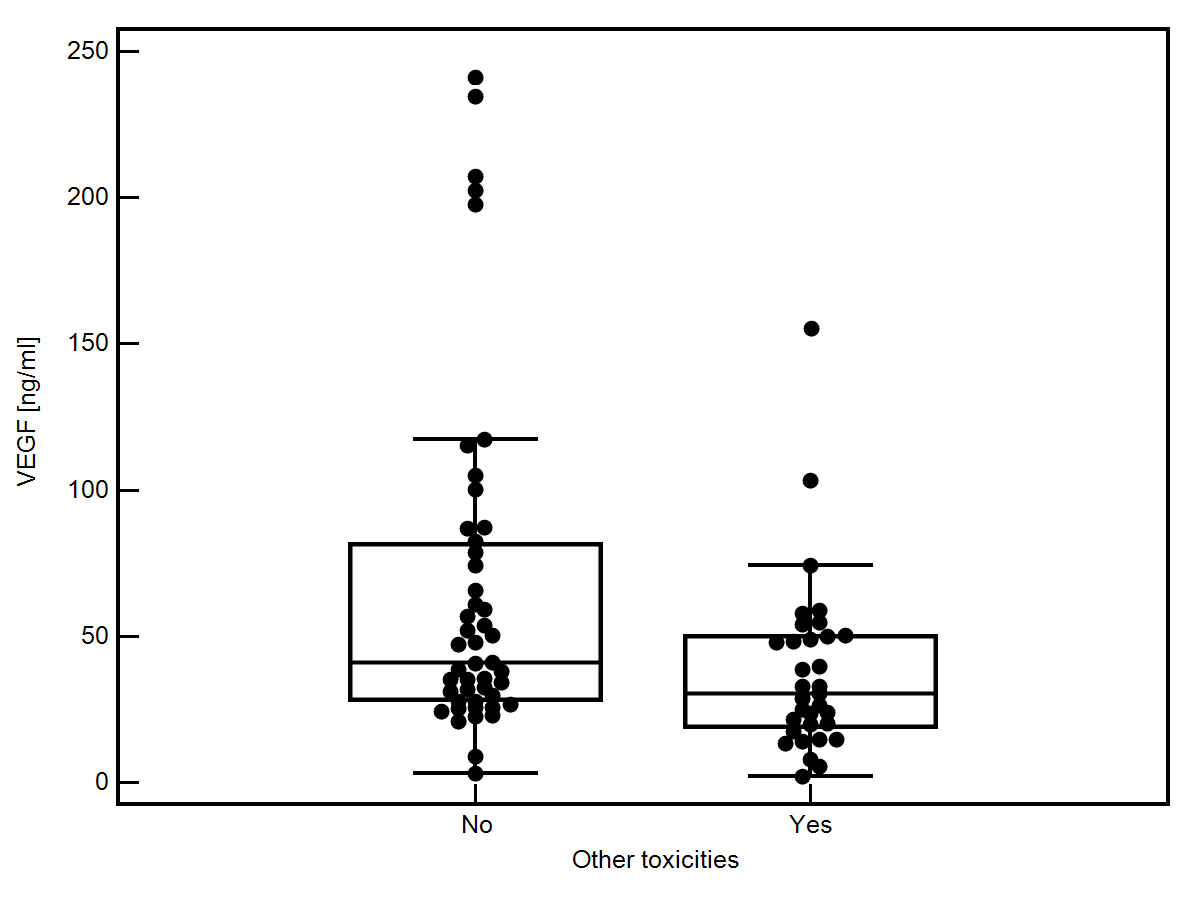


Abbreviations: VEGF, vascular endothelial growth factor

**Supplementary Figure 3**. Comparison of the concentration of VEGF depending on the presence of other toxicities; MedCalc 15.8 PL (MedCalc Software Ltd, Belgium)
